# Supplementary material for: High levels of infectiousness of asymptomatic Leishmania (Viannia) braziliensis infections in wild rodents highlights their importance in the epidemiology of American Tegumentary Leishmaniasis in Brazil
Source: PLoS Negl Trop Dis. 2023 Jan 30;17(1):e0010996. doi: 10.1371/journal.pntd.0010996 (PMC9910795; doi:10.1371/journal.pntd.0010996)
Supplement: S1 Table — (DOCX) [file pntd.0010996.s007.docx]

S1 Table. Trapping schedule showing the number of trap-nights (total single traps set over trapping period)

| Date range | Trap round | Trip Duration | Nights  Trapping | Peri domestic | Plantation | Forest |
| --- | --- | --- | --- | --- | --- | --- |
| 22/05 to28/05/12 | 1 | 7 | 5 | 25 | 275 | 100 |
| 27/06 to 01/07/12 | 2 | 6 | 5 | 25 | 275 | 100 |
| 28/07 to 01/09/12 | 3 | 6 | 5 | 25 | 275 | 100 |
| 17/10 to 20/10/12 | 4 | 4 | 4 | 20 | 220 | 80 |
| 27/11 to 30/11/12 | 5 | 4 | 4 | 20 | 220 | 80 |
| 29/01 tp 06/02/13 | 6 | 9 | 9 | 45 | 495 | 180 |
| 19/03 to 26/03/13 | 7 | 8 | 9 | 40 | 440 | 160 |
| 09/04 to 17/04/13 | 8 | 9 | 9 | 45 | 495 | 180 |
| 04/06 to 12/06/13 | 9 | 9 | 8 | 45 | 495 | 180 |
| 09/07 to 11/07/13 | 10 | 3 | 3 | 15 | 165 | 60 |
| 20/08 to 29/08/13 | 11 | 10 | 6 | 40 | 440 | 160 |
| 10/09 to 19/09/13 | 12 | 10 | 8 | 40 | 440 | 160 |
| 08/10 to 18/10/13 | 13 | 11 | 7 | 35 | 385 | 140 |
| 19/11 to 28/11/13 | 14 | 10 | 8 | 40 | 440 | 160 |
| 10/12 to 19/12/13 | 15 | 10 | 8 | 40 | 440 | 160 |
| 21/01 to 30/01/14 | 16 | 10 | 8 | 40 | 440 | 160 |
| 27/03 to 28/03/14 | 17 | 2 | 2 | 10 | 110 | 40 |
| 20/05 to 24/05/14 | 18 | 5 | 5 | 25 | 275 | 100 |
| 01/07 to 05/07/14 | 19 | 5 | 5 | 25 | 275 | 100 |
| 29/07 to 01/08/14 | 20 | 4 | 4 | 20 | 220 | 80 |
| Total |  | 142 | 124 | 620 | 6820 | 2480 |
