# Supplementary material for: High levels of infectiousness of asymptomatic Leishmania (Viannia) braziliensis infections in wild rodents highlights their importance in the epidemiology of American Tegumentary Leishmaniasis in Brazil
Source: PLoS Negl Trop Dis. 2023 Jan 30;17(1):e0010996. doi: 10.1371/journal.pntd.0010996 (PMC9910795; doi:10.1371/journal.pntd.0010996)
Supplement: S2 Table — (DOCX) [file pntd.0010996.s008.docx]

S2 Table. Median recapture interval (days) and number of times (re)captured by species

| Rodent species | recapture interval (days) | | | number of times individuals recaptured | | |
| --- | --- | --- | --- | --- | --- | --- |
|  | n recaptures | median (IQR) | range | n individuals | median (IQR) | range |
| *Akodon arviculoides* | 5 | 49 (32.5, 153.5) | 16-183 | 32 | 1 (1, 2.5) | 1-3 |
| *Holochillus sciureus* | 22 | 69 (41.0, 132.3 | 20-264 | 40 | 1 (1, 2) | 1-4 |
| *Necromys lasiurus* | 30 | 48 (33.8, 95.3) | 16-193 | 83 | 1 (1, 2) | 1-4 |
| *Nectomys squamipes* | 279 | 45 (33.0, 80.0) | 16-391 | 245 | 2 (1, 3) | 1-11 |
| *Olygoryzomys eliurus* | 0 | - | - | 2 | 0 | - |
| *Oryzomys subflavus* | 0 | - | - | 4 | 0 | - |
| *Oxymycterus angulares* | 25 | 63 (35.5, 84.0) | 16-203 | 49 | 1 (1, 2.5) | 1-4 |
| *Rattus rattus* | 36 | 68 (41.0, 122.8) | 16-456 | 148 | 1 (1, 1) | 1-3 |
