# Supplementary material for: High levels of infectiousness of asymptomatic Leishmania (Viannia) braziliensis infections in wild rodents highlights their importance in the epidemiology of American Tegumentary Leishmaniasis in Brazil
Source: PLoS Negl Trop Dis. 2023 Jan 30;17(1):e0010996. doi: 10.1371/journal.pntd.0010996 (PMC9910795; doi:10.1371/journal.pntd.0010996)
Supplement: S3 Table — (DOCX) [file pntd.0010996.s009.docx]

S3 Table. Infection prevalences based on conventional PCR compared to quantitative PCR (qPCR) of rodent blood samples using PCR target primers specific to parasites of the genus *Leishmania* (*Viannia*) *braziliensis*.

| Species | PCR pos/total (proportion) | qPCR pos/total (proportion) |
| --- | --- | --- |
| *Akodon arviculoides* | 0/37 (0) | 5/34 (0.147) |
| *Holochillus sciureus* | 14/62 (0.226) | 21/60 (0.350) |
| *Necromys lasiurus* | 0/113 (0) | 34/112 (0.304) |
| *Nectomys squamipes* | 41/523 (0.078) | 254/513 (0.495) |
| *Olygoryzomys eliurus* | 0/2 (0) | 0/2 (0) |
| *Oryzomys subflavus* | 0/4 (0) | 0/4 (0) |
| *Oxymycterus angulares* | 1/74 (0.014) | 21/74 (0.284) |
| *Rattus rattus* | 2/184 (0.011) | 47/184 (0.255) |
| Total | 58/999 (0.058) | 382/983 (0.389) |
