# Supplementary material for: High levels of infectiousness of asymptomatic Leishmania (Viannia) braziliensis infections in wild rodents highlights their importance in the epidemiology of American Tegumentary Leishmaniasis in Brazil
Source: PLoS Negl Trop Dis. 2023 Jan 30;17(1):e0010996. doi: 10.1371/journal.pntd.0010996 (PMC9910795; doi:10.1371/journal.pntd.0010996)
Supplement: S4 Table — (DOCX) [file pntd.0010996.s010.docx]

S4 Table. Infection and infectiousness of individual rodents at the time of xenodiagnosis, and the associated *L.* (*Viannia*) *braziliensis* parasite loads in the rodent blood and xenopositive *Nyssomyia whitmani* sand flies.

| Rodent species | Individual ID | Days since 1st capture | Rodent qPCR/ PCR positive | Number xeno positive flies | Number flies exposed | Prop. flies positive | *Leishmania* load in 200µL of blood | *Leishmania* load per xeno positive fly |
| --- | --- | --- | --- | --- | --- | --- | --- | --- |
| *Akodon arviculoides* | 572 | 124 | 1 | 6 | 9 | 0.657 | 25 | 19 |
| *Necromys lasiurus* | 573 | 61 | 1 | 11 | 25 | 0.447 | 77 | 6 |
| *Nectomys squamipes* | 5 | 610 | 1 | 0 | 12 | 0.000 | 83 | 0 |
| *Nectomys squamipes* | 40 | 565 | 0 | 1 | 11 | 0.060 | 0 | 2 |
| *Nectomys squamipes* | 40 | 608 | 1 | 12 | 52 | 0.231 | 39 | 2 |
| *Nectomys squamipes* | 272 | 175 | 1 | 4 | 10 | 0.394 | 0 | 22 |
| *Nectomys squamipes* | 379 | 282 | 1 | 24 | 93 | 0.258 | 46 | 30 |
| *Nectomys squamipes* | 387 | 168 | 0 | 1 | 26 | 0.051 | 0 | 7 |
| *Nectomys squamipes* | 393 | 194 | 0 | 4 | 6 | 0.657 | 0 | 120 |
| *Nectomys squamipes* | 393 | 394 | 1 | 13 | 25 | 0.526 | 17 | 8 |
| *Nectomys squamipes* | 393 | 295 | 1 | 4 | 18 | 0.219 | 35 | 1 |
| *Nectomys squamipes* | 397 | 347 | 1 | 16 | 24 | 0.657 | 10 | 65 |
| *Nectomys squamipes* | 398 | 391 | 1 | 10 | 15 | 0.657 | 2 | 8 |
| *Nectomys squamipes* | 404 | 188 | 0 | 2 | 16 | 0.123 | 0 | 118 |
| *Nectomys squamipes* | 404 | 231 | 1 | 2 | 22 | 0.090 | 40 | 2 |
| *Nectomys squamipes* | 410 | 229 | 1 | 1 | 23 | 0.029 | 29 | 2 |
| *Nectomys squamipes* | 421 | 191 | 0 | 0 | 23 | 0.000 | 0 | 0 |
| *Nectomys squamipes* | 421 | 292 | 1 | 0 | 18 | 0.000 | 45 | 0 |
| *Nectomys squamipes* | 432 | 224 | 1 | 5 | 43 | 0.122 | 738 | 15 |
| *Nectomys squamipes* | 432 | 343 | 1 | 10 | 16 | 0.616 | 7 | 8 |
| *Nectomys squamipes* | 432 | 413 | 0 | 2 | 6 | 0.329 | 0 | 1 |
| *Nectomys squamipes* | 432 | 384 | 1 | 12 | 19 | 0.657 | 54 | 58 |
| *Nectomys squamipes* | 452 | 385 | 1 | 31 | 48 | 0.643 | 1 | 176 |
| *Nectomys squamipes* | 452 | 357 | 1 | 1 | 1 | 0.657 | 18 | 52 |
| *Nectomys squamipes* | 452 | 316 | 1 | 22 | 34 | 0.638 | 1 | 501 |
| *Nectomys squamipes* | 456 | 274 | 1 | 20 | 30 | 0.657 | 12 | 155 |
| *Nectomys squamipes* | 505 | 253 | 1 | 35 | 54 | 0.645 | 15 | 192 |
| *Nectomys squamipes* | 524 | 243 | 1 | 19 | 34 | 0.560 | 5 | 10 |
| *Nectomys squamipes* | 524 | 28 | 1 | 3 | 4 | 0.657 | 3 | 63 |
| *Nectomys squamipes* | 567 | 181 | 1 | 20 | 34 | 0.599 | 7 | 33 |
| *Nectomys squamipes* | 577 | 217 | 1 | 22 | 35 | 0.638 | 0 | 76 |
| *Nectomys squamipes* | 607 | 70 | 1 | 19 | 33 | 0.577 | 3 | 2 |
| *Nectomys squamipes* | 618 | 0 | 1 | 51 | 81 | 0.625 | 13 | 42 |
| *Nectomys squamipes* | 618 | 40 | 1 | 60 | 92 | 0.657 | 4 | 8 |
| *Nectomys squamipes* | 619 | 0 | 1 | 19 | 29 | 0.657 | 18 | 17 |
| *Nectomys squamipes* | 623 | 69 | 1 | 206 | 318 | 0.649 | 1 | 86 |
| *Nectomys squamipes* | 633 | 0 | 1 | 6 | 9 | 0.657 | 2 | 269 |
| *Nectomys squamipes* | 634 | 0 | 0 | 4 | 6 | 0.657 | 0 | 15 |
| *Nectomys squamipes* | 635 | 0 | 0 | 45 | 69 | 0.657 | 0 | 55 |
| *Nectomys squamipes* | 636 | 0 | 1 | 9 | 14 | 0.610 | 235 | 12 |
| *Nectomys squamipes* | 637 | 0 | 1 | 5 | 8 | 0.575 | 0 | 2 |
| *Oxymycterus angulares* | 221 | 353 | 1 | 1 | 45 | 0.015 | 74 | 3 |
| *Rattus rattus* | 443 | 196 | 1 | 12 | 18 | 0.657 | 364 | 19 |
| *Rattus rattus* | 501 | 133 | 1 | 9 | 14 | 0.610 | 90 | 25 |
