# Supplementary material for: High levels of infectiousness of asymptomatic Leishmania (Viannia) braziliensis infections in wild rodents highlights their importance in the epidemiology of American Tegumentary Leishmaniasis in Brazil
Source: PLoS Negl Trop Dis. 2023 Jan 30;17(1):e0010996. doi: 10.1371/journal.pntd.0010996 (PMC9910795; doi:10.1371/journal.pntd.0010996)
Supplement: S5 Table — (DOCX) [file pntd.0010996.s011.docx]

S5 Table. Infection and infectiousness of individual rodents at the time of xenodiagnosis, and the associated *L.* (*V*.) *braziliensis* parasite loads in the rodent blood and xenopositive *Lutzomyia longipalpis* sand flies.

| Rodent species | Individual ID | Days since 1st capture | qPCR/ PCR (pos/neg) | Xeno n positive flies | Xeno N exposed flies | Prop. flies positive | *Leishmania* load in 200µL of blood | *Leishmania* load per xeno positive fly |
| --- | --- | --- | --- | --- | --- | --- | --- | --- |
| *Nectomys squamipes* | 12 | 189 | 1 | 9 | 82 | 0.104 | 447 | 50 |
| *Nectomys squamipes* | 16 | 189 | 1 | 17 | 58 | 0.295 | 606 | 295 |
| *Nectomys squamipes* | 185 | 67 | 0 | 47 | 80 | 0.583 | 0 | 10 |
| *Rattus rattus* | 187 | 67 | 1 | 28 | 53 | 0.521 | 10 | 111 |
| *Rattus rattus* | 188 | 67 | 1 | 64 | 134 | 0.480 | 9 | 23 |
