# Supplementary material for: High levels of infectiousness of asymptomatic Leishmania (Viannia) braziliensis infections in wild rodents highlights their importance in the epidemiology of American Tegumentary Leishmaniasis in Brazil
Source: PLoS Negl Trop Dis. 2023 Jan 30;17(1):e0010996. doi: 10.1371/journal.pntd.0010996 (PMC9910795; doi:10.1371/journal.pntd.0010996)
Supplement: Supplementary file 9 [file pntd.0010996.s006.docx]

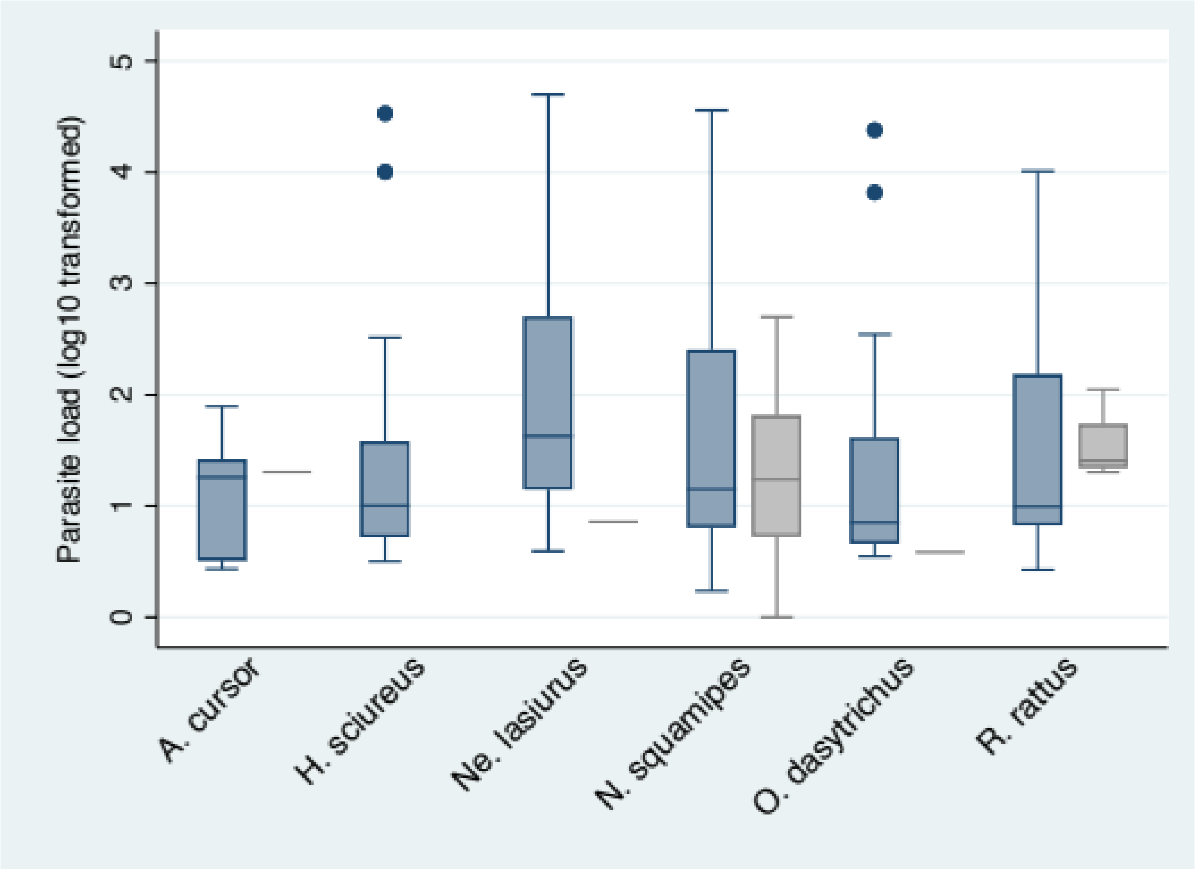
Parasite load (log10 transformed)
0
1
2
3
4
5
A. cursor
H. sciureus
Ne. lasiurus
N. squamipes
O. dasytrichus
R. rattus
