# Supplementary material for: High levels of infectiousness of asymptomatic Leishmania (Viannia) braziliensis infections in wild rodents highlights their importance in the epidemiology of American Tegumentary Leishmaniasis in Brazil
Source: PLoS Negl Trop Dis. 2023 Jan 30;17(1):e0010996. doi: 10.1371/journal.pntd.0010996 (PMC9910795; doi:10.1371/journal.pntd.0010996)
Supplement: Attachment — Submitted filename: PNTD-D-22-00244 Reply to reviewers.doc [file pntd.0010996.s012.doc]

The manuscript by Marinho-Junior et al presents a comprehensive reservoir collection capture that provide important information for the specialized Leishmania/sand fly community. Presentation and discussion of the data should be optimized in order to favor the manuscript:

1) Points raised by the referees should be answered carefully.

2) The data comparison blood vs. skin qPCR is not helping the discussion and presentation interpretation of the most important information present in the manuscript: the reservoirs. I suggest the authors combine the positivity of results to create a cut-off of positivity calling radar than compare the blood vs biopsy values. The skin location my not be adequate for parasite search and its extension as well not. This particular data comparison detract from the main point of the paper. Due that, Fig 4 is not helping and can be combined with S6 if authors want to keep.

*The point made by the referee is extremely pertinent and we agree entirely and have deleted Fig. 4 and have decided not to combine it with Fig.S6.*

*3) Infectiousness to sand flies should be better explained.*

Why the significant variation in the number of flies used for the xenodiagnostic?

*The maximum number flies at the time of the capture were used but varied according to the number of wild flies captured and availability of laboratory bred longipalpis.*

As suggestion to improve the manuscript readability, the authors should consider:

4) A shorter title.

*Title shortend.*

5) (line32) "infectiousness to" to "infectiousness potential to". It is more accurate to the data.

*Inserted*

6) The biology of sand flies multiple blood meals could help the manuscript interpretation. It's a pivotal biology fact of this insect vector that should not be neglected on this manuscript discussion.

*Agreed and have inserted a relevant text*

7) lines 118-122. This entire paragraph is not accurate to literature available. Is highly speculative and not helping the manuscript main point.

*Paragraph deleted*

8) Conclusions are important and adequate. The discussion should be simplified to match that.

*Discussion simplified where authors felt it feasible*

Reviewer's Responses to Questions

<b>Key Review Criteria Required for Acceptance?</b></br></br>

As you describe the new analyses required for acceptance, please consider the following:</br></br>

<b>Methods</b></br></br>

-Are the objectives of the study clearly articulated with a clear testable hypothesis stated?</br>

-Is the study design appropriate to address the stated objectives?</br>

-Is the population clearly described and appropriate for the hypothesis being tested?</br>

-Is the sample size sufficient to ensure adequate power to address the hypothesis being tested?</br>

-Were correct statistical analysis used to support conclusions?</br>

-Are there concerns about ethical or regulatory requirements being met?</br></br>

Reviewer #1: The authors have met all method criteria to have the manuscript accepted.

Reviewer #2: It is not clear how the exposure of the captured rodents to wild infected sand flies impact in posterior prevalence when they are recaptured. Unless it is a misunderstanding, I think this is a serious problem in the design of this manuscript. It is also not clear how this was considered in terms of ethical use of research animals. Besides that, this problem is specially important if we consider the recent findings of Valenzuela´s group about the change in Leishmania development and differentiation after a second blood meal. It is impossible to know the proportion of sand flies that might have been considered not-infected in the control screening but appear as infected in the xenodiagnosis due to amplification of parasite populations.

*The study was designed to show that over a period of time rodents remain infected and are infectious to sand flies and not how it impacts the future prevalence. We agree that Velenzuela’s group showed that a second blood meal enhanced subsequent development but consider that the qPCR detects such infections mentioned by the reviewer.*

Reviewer #3: Most of the study is well designed, and the methods are clearly and thoroughly described. The sample of 603 rodents, captured/recaptured in 20 trapping rounds over two years (9.920 single trap nights) is sufficient to show rodent species diversity and infection prevalence in three study habitats. Statistical analyses are well performed and well described.

The only problematic method is the use of wild-caught Ny. whitmani females for xenodiagnosis. To calculate infection rates and parasite loads in xenodiagnoses, authors subtracted the corresponding values for wild-caught flies from the experimental data. Therefore, the resulting data may be affected by unequal representation of naturally infected flies among sand fly groups applied to individual animals. Also, parasite loads in naturally infected flies with mature infection would be much higher than in freshly engorged females during the experiment. Fortunately, the authors also used L. longipalpis flies from the colony in 5 experiments and these experiments did not yield substantially different results (Table 7, S4 and S5). Above mentioned facts should be emphasized in the Discussion and the Abstract: xenodiagnoses results gained by wild-caught sand flies should be treated with extreme caution and exclusively sand flies from the colony should be used for future studies.

*The infection rate in wild caught whitmani was accounted for in calculating the infection levels found in the experimentally wild caught flies. As the reviewer noted the comparison of the rates in whitmani and longipalpis indicates that this correction was correct. We agree with the referee and have included wording in accordance with this.*

--------------------

<b>Results</b></br></br>

-Does the analysis presented match the analysis plan?</br>

-Are the results clearly and completely presented?</br>

-Are the figures (Tables, Images) of sufficient quality for clarity?</br></br>

Reviewer #1: Yes, the analysis presented match the analysis plan and the results are clearly and completely presented. The tables and figures are of sufficient quality; however, photos and Google map image of the region are redundant and should be excluded, since methods describe in detail the areas of study.

Reviewer #2: The analysis is straightforward, the results and figures are well presented.

Reviewer #3: Results are clearly presented, and all the figures and tables are of sufficient quality (minor suggestions are in the section Editorial Modifications).

--------------------

<b>Conclusions</b></br></br>

-Are the conclusions supported by the data presented?</br>

-Are the limitations of analysis clearly described?</br>

-Do the authors discuss how these data can be helpful to advance our understanding of the topic under study?</br>

-Is public health relevance addressed?</br></br>

Reviewer #1: Yes, the conclusions are supported by the data presented, limitation of analysis particularly Xenodiagnoses using field collected Ny. whitmani were justified and the authors discuss how their findings can be helpful to advance our understanding of the topic under study. The manuscript has an important public health relevance; however, the authors should have clearly stated in the discussion how their findings can help public health authorities in Brazil to prevent the spread of Leishmania (Viannia) braziliensis in the endemic area.

Reviewer #2: The results are relevant in a very broad and qualitative sense. However, the impact of the exposure to wild infected sand flies in my opinion clearly impacts the reliability of the quantitative results.

Reviewer #3: See the Summary and General Comments section.

--------------------

<b>Editorial and Data Presentation Modifications?</b></br><br/>

Use  this section for editorial suggestions as well as relatively minor modifications of existing data that would enhance clarity. If the only modifications needed are minor and/or editorial, you may wish to recommend “Minor Revision” or “Accept”.

Reviewer #1: Minor modifications are needed in order to address typo mistake and presence of figures irrelevant for the understanding of the manuscript.

Reviewer #2: (No Response)

Reviewer #3: Table 1, 6 and S1: “Peridomestic” or “domestic” should be replaced with “Peri domestic”

*These have been dealt with in the relevant tables and text*

Figure S2 can be omitted because it does not provide more information than Figure S3 (however, since it is included in the supplementary material, redundancy may not be an issue).

*We prefer to keep Fig. S1 and Fig. S3. The referee considers that Fig S3 does not contain more information than Fig. S1, however we want to keep it as it gives a visual idea of the homesteads and differences in the proximity of plantations.*

Line 93: Are authors sure these two non-English literary sources should be cited here (2, 3)?

*They are included as they refer specifically to Latin American locations where there is expansion in peri domestic environments which are similar to the study site. For this reason, we wish to include them.*

Line 114: The mentioned few experimental studies like de Moura et al (2005, doi.org/10.1128/IAI.73.9.5827-5834.2005) should be cited.

*This citation has been included*

Table 1: Ol. nigripes

*Corrected*

Lines 258-267: Negative control should be specified for qPCR.

*Included on line 262.*

Line 332: According to Fig S4b, H. sciureus and O. dasytrichus have been recaptured up to 5 times.

*Line 332 corrected to 5 times*

Table 3: The blood volume 0.2 mL (not 0.2µL)

*Corrected to 0.2 mL*

Discussion on lines 541-546: The study “Christensen and Herrer 1972 (TRANS R SOC TROP MED HYG 66(5))” showing the infectiousness of Choloepus hoffmanni infected with L braziliensis to vectors should also be cited.

Reference and additional text included

Discussion on lines 631-638: The authors should add to the discussion that another reason for the higher positivity values of the tissue samples from the ears in the laboratory study may be that in this field study only half the tissue sample was taken (25 vs 50 mg)

*Comment added to the above effect.*

--------------------

<b>Summary and General Comments</b></br></br>

Use  this section to provide overall comments, discuss strengths/weaknesses of the study, novelty, significance, general execution and scholarship. You may also include additional comments for the author, including concerns about dual publication, research ethics, or publication ethics. If requesting major revision, please articulate the new experiments that are needed.

Reviewer #1: Dear editor of Plos. Neglected Tropical Diseases,

I would like to thank you very much for the opportunity to review the manuscript of Dr. Marinho-Junior et al. PNTD-D-22-00244- “Chronic asymptomatic infection associated with high levels of infectiousness of wild rodent communities highlights their role in the epizootiology of human American Tegumentary Leishmaniasis: a capture-mark-recapture study in Brazil.

Apart from a small typo mistake in line 48, the paper is very well written and, in my view, meets the proper usage of English language standards of Plos, Neglected Tropical Diseases, granting it merits for publication.

Briefly, the authors studied the epidemiological significance of wildlife infections among guild of rodent reservoirs present in three distinct ecotopes in two municipalities in the city of Amaraji, State of Pernambuco, Brazil, for the presence of Leishmania (Viannia) braziliensis by employing capture-remake-recapture (CMR), traditional PCR, qPCR and xenodiagnosis methods aiming to address five different goals. The results show a highly interesting and unique overview of the dispersion of infected putative rodent reservoirs among the three distinct ecotopes with the participation of the natural vector Ny. whitmani. The data presented can assist local public health authorities in determining the correct strategies for reducing the transmission of L.(V) brazilinesis in the region, which confirms the high relevance and scientific contribution of the manuscript.

According to my interpretation of the discussion the authors seem to believe in independent transmission cycles occurring in each ecotope, considering the free movement of reservoirs, hosts and vector(s) in the region, which might help understand what happens to parasite and vector(s) during the non (low) and high transmission periods. This alone is a novel approach to the traditional eco-epidemiology of Leishmaniasis and I applaud the authors for it.

A few comments that I would like to make, without diminishing the extremely laborious and time-consuming field and laboratory work of the authors, are:

1- The authors should have included a table containing the sand fly fauna present in each ecotope, although not in the five main goals of the paper the distribution, frequency and seasonality of sand fly species per ecotope would have enriched the manuscript.

*Clearly, we agree but this is another study that was impossible due to logistical difficulties (Finance and staff).*

2- In addition, it would have been extremely valuable if the authors had collected the most abundant sand fly species of each ecotope (probably Ny. whitmani) and scrutinized their blood meal content before using some sand flies for xenodiagnoses. It is well known now that a second and successive blood meals augment the parasite infectivity in the sand fly, at least in laboratory conditions, please refer to the work of Serafim et al, Nat Microbiol. 2018. Also, the authors might be able to evaluate the reservoir preference of Ny.whitmani with the three most abundant rodent species present in the three distinct ecotopes, if blood meals were analyzed.

*Again we agree with the author and this is a study that we plan in the future if funds become available. We also agree upon the important implications of Serafim et al but do not think it is relevant to the present paper whose focus is infectiousness.*

2- Another highly desired approach, would have been for the authors to bring Ny, whitman to the lab and rear the species for one generation, since it seems to be extremely difficult to establish a Ny. whitmani colony in captivity, before taking some flies back to the filed for feeding on the three most abundant rodent reservoir species. This, relatively simple method, would have shed some light on the role of Ny. whitmani as the main vector in the area..

*Attempts so far to rear Ny. whitmani in our laboratories have failed. But we plan to continue trying and are hoping for a breakthrough.*

3- The 6% naturally infected sand flies observed during standardization of xenodiagnoses to count for potential Leishmania background level, since authors initially used field collected Ny. whitmani, seems to be extremely high considering other field studies in Brazil. Did the authors collect the flies from the ecotope where most of infected reservoirs were present (plantations) or authors just did a pool containing flies from all three ecotypes? Please, clarify it in the methods.

*Ny. whitmani was not collected where the mammal trapping was performed. They were captured as is cited under the heading* ***Xenodiagnosis:***

*Xenodiagnosis was performed using wild Ny. whitmani sand flies which were trapped by CDC light traps (set 6pm-6am) in animal shelters, stables and chicken coops in the nearby locality of Engenho Raiz Nova where Ny. whitmani is the dominant sand fly species [23].*

*It was considered that any trapping of flies where the animals were captured could potentially change the transmission and parasitemias which was what we were following in recaptured animals.*

4- Why did the authors not attempt to transmit L(V) braziliensis to hamsters in the lab by the bite of Ny. whitmani used for xenodiagnoses? Did the authors try to maintain Ny. whitmani in the lab after xenodiagnoses for observing in vivo parasite differentiation and development? It would have demonstrated the degree of infectivity and transmissibility of this vector, satisfying the criteria proposed by Killick-Kendrick to incriminate a sand fly as a vector.

*Again, we agree that this would be excellent but it was logistically not possible given that we were concentrating on xenodiagnosis and wanted to use all the available flies for this.*

5- I assume that during editing of the paper lines 343, 380,387, 431,465 and 513 will be deleted.

*We assume the same*

6- I don’t think it is necessary to include the Google map location and photos of the area, since the localities are very well described in the methods.

*We agree that the localities are well described in the methods but wish to include the maps and photos to facilitate the reader. Also these are supplementary Figures so they are not a burden to the manuscript*.

Sincerely,

Referee A

Reviewer #3: The manuscript elucidated the reservoir system of L. braziliensis in Pernambuco, NE Brazil. The authors studied rodent communities in three interconnected habitats and described the involvement of each rodent species in the transmission of the parasite, with a precise description of their habitat association and infection risk in different habitats. Long-term maintenance of infections was documented by recapture of infected animals and their persistent infectiousness to vectors using xenodiagnosis. The manuscript is very well written; it is based on a sufficient amount of material that is perfectly statistically evaluated and presented. I suggest minor revision of the manuscript (see my comment about xenodiagnoses with wild-caught sand flies).

--------------------

PLOS authors have the option to publish the peer review history of their article (<a href="https://journals.plos.org/plosntds/s/editorial-and-peer-review-process#loc-peer-review-history" target="_blank">what does this mean?</a>). If published, this will include your full peer review and any attached files.<br><br>

If you choose “no”, your identity will remain anonymous but your review may still be made public.<br><br>

<b>Do you want your identity to be public for this peer review?</b> For information about this choice, including consent withdrawal, please see our <a href="https://www.plos.org/privacy-policy" target="_blank">Privacy Policy</a>.

Reviewer #1: No

Reviewer #2: No

Reviewer #3: No

Figure Files:

While revising your submission, please upload your figure files to the Preflight Analysis and Conversion Engine (PACE) digital diagnostic tool, https://pacev2.apexcovantage.com. PACE helps ensure that figures meet PLOS requirements. To use PACE, you must first register as a user. Then, login and navigate to the UPLOAD tab, where you will find detailed instructions on how to use the tool. If you encounter any issues or have any questions when using PACE, please email us at figures@plos.org.

Data Requirements:

Please note that, as a condition of publication, PLOS' data policy requires that you make available all data used to draw the conclusions outlined in your manuscript. Data must be deposited in an appropriate repository, included within the body of the manuscript, or uploaded as supporting information. This includes all numerical values that were used to generate graphs, histograms etc.. For an example see here: http://www.plosbiology.org/article/info%3Adoi%2F10.1371%2Fjournal.pbio.1001908#s5.

Reproducibility:

To enhance the reproducibility of your results, we recommend that you deposit your laboratory protocols in protocols.io, where a protocol can be assigned its own identifier (DOI) such that it can be cited independently in the future. Additionally, PLOS ONE offers an option to publish peer-reviewed clinical study protocols. Read more information on sharing protocols at https://plos.org/protocols?utm_medium=editorial-email&utm_source=authorletters&utm_campaign=protocols

__________________________________________________

In compliance with data protection regulations, you may request that we remove your personal registration details at any time.  (Use the following URL: https://www.editorialmanager.com/pntd/login.asp?a=r). Please contact the publication office if you have any questions.
